# Jinmaitong Alleviates Diabetic Neuropathic Pain Through Modulation of NLRP3 Inflammasome and Gasdermin D in Dorsal Root Ganglia of Diabetic Rats

**DOI:** 10.3389/fphar.2021.679188

**Published:** 2021-11-03

**Authors:** Qing Sun, Rui Zhang, Xiaowei Xue, Qunli Wu, Dan Yang, Chao Wang, Bin Yan, Xiaochun Liang

**Affiliations:** ^1^ Department of Traditional Chinese Medicine, Peking Union Medical College Hospital, Peking Union Medical College, Chinese Academy of Medical Sciences, Beijing, China; ^2^ Department of Laboratory Medicine, Peking Union Medical College Hospital, Peking Union Medical College, Chinese Academy of Medical Sciences, Beijing, China; ^3^ Department of Pathology, Peking Union Medical College Hospital, Peking Union Medical College, Chinese Academy of Medical Sciences, Beijing, China; ^4^ Department of Integrative Oncology, China-Japan Friendship Hospital, Beijing, China

**Keywords:** traditional Chinese medicine, pyroptosis, NLRP3 inflammasome, jinmaitong, diabetic neuropathic pain

## Abstract

Jinmaitong (JMT) is a compound prescription of traditional Chinese medicine that has been used to treat diabetic neuropathic pain (DNP) for many years. Here, we investigated the effects of JMT on the activation of NOD-like receptor family pyrin domain-containing 3 (NLRP3) inflammasome and pyroptosis in Dorsal root ganglia (DRG) of diabetic rats. Streptozotocin (STZ)-induced diabetic rats were gavaged with JMT (0.88 g/kg/d) or alpha-lipoic acid (ALA, positive control, 0.48 mmol/kg/d) for 12 weeks. Distilled water was administered as a vehicle control to both diabetic and non-affected control rats. Blood glucose levels and body weights were measured. Behavioral changes were tested with mechanical withdrawal threshold (MWT) and tail-flick latency (TFL) tests. Morphological injury associated with DRG was observed with hematoxylin and eosin (H&E) and Nissl’s staining. mRNA and protein levels of NLRP3 inflammasome components (NLRP3, ASC, caspase-1), downstream IL-1β and gasdermin D (GSDMD) were evaluated by immunohistochemistry, quantitative real time-PCR and western blot. The results showed that JMT had no effect on blood glucose levels and body weights, but significantly improved MWT and TFL behavior in diabetic rats, and attenuated morphological damage in the DRG tissues. Importantly, JMT decreased the mRNA and protein levels of components of NLRP3 inflammasome, including NLRP3, ASC and caspase-1. JMT also down-regulated the expression of IL-1β and GSDMD in the DRG of DNP rats. In addition, ALA treatment did not perform better than JMT. In conclusion, JMT effectively relieved DNP by decreasing NLRP3 inflammasome activation and pyroptosis, providing new evidence supporting JMT as an alternative treatment for DNP.

## Introduction

Peripheral neuropathy is the most prevalent diabetes-associated complication with an overall prevalence of 50–60% (Pop-Busui et al., 2017). Diabetic neuropathic pain (DNP) is a common type of diabetic peripheral neuropathy, accounting for approximately 25% of patients, which occurs in the early stage of diabetes and even in the stages of impaired glucose tolerance (Davies et al., 2006). DNP is caused by small nerve fibers with clinical manifestations that include allodynia, hyperalgesia and spontaneous pain. Symptoms of chronic DNP can last for years and can severely impair quality of life (Benbow et al., 1998), leading to anxiety, depression and other psychological problems. This results in a substantial economic and social burden for patients experiencing DNP. Currently, stable glycemic control and pain management are the only modifiable treatments for DNP, calling for investigation into new, more effective treatments (Feldman et al., 2017).

While several etiological theories of DNP have emerged, inflammation-mediated neuronal and peripheral glial cell damage has been discovered to drive DNP pathogenesis (Pop-Busui et al., 2016). Recent studies investigated NOD-like receptor family pyrin domain-3 (NLRP3) inflammasome-mediated inflammation and pyroptosis in diabetes and its complications (Schroder et al., 2010; Yu et al., 2020; Sharma and Kanneganti, 2021). NLRP3 inflammasome is a multiprotein complex that coordinates innate immune responses by activating caspase-1 and inducing the pro-inflammatory cytokines interleukin (IL) -1β and IL-18 (Martinon et al., 2002; Broz and Dixit, 2016). Activated caspase-1 cleaves the linker between the N-terminal and C-terminal domains of gasdermin D (GSDMD). The N-terminal domain then binds to phosphoinositides on the plasma membrane and generates membrane pores, thus inducing pyroptosis and the secretion of proinflammatory IL-1β (Shi et al., 2015; Ding et al., 2016; Liu et al., 2016). GSDMD is vital for pyroptosis execution and interleukin-1β secretion (He et al., 2015).

Dorsal root ganglia (DRG) neurons, primary sensory neurons whose somas bundle together in DRG to impose extreme metabolic demands, are particularly vulnerable to toxins and systemic metabolic derangements (McHugh and McHugh, 2004). Previous studies found that NLRP3 inflammasome activation and downstream DRG inflammation led to painful neuropathy, lumbar disc herniation, and bortezomib-induced mechanical allodynia in type 2 diabetic rodents (Jin et al., 2017; Zhang et al., 2017; Liu et al., 2018; Thakur et al., 2020). However, the specific mechanism of NLRP3 inflammasome-induced pyroptosis in DRG remains unclear in the context of DNP.

Jinmaitong (JMT), a compound prescription of traditional Chinese medicine (TCM), has been used to prevent and treat DNP for several decades. Our randomized double-blind clinical study demonstrated that JMT not only ameliorated chronic limb pain and numbness, but also improved blood glucose levels, lipid metabolism, and nerve conduction velocity (NCV) in DNP patients (Liang et al., 1999). Experimental evidence indicates that JMT rescues mechanical allodynia and morphological injury in the DRG of diabetic rats, likely by suppressing niacinamide adenine dinucleotide phosphoric acid (NADPH) oxidase activation and mitochondrial-mediated apoptosis (Liu et al., 2013). High glucose induces inflammation and apoptosis through Nf-E2 related factor 2 (Nrf2)/heme oxygenase-1 (HO-1) activation and nuclear factor-κappa B (NF-κB) inhibition *in vitro*; several chemical compounds extracted from JMT, including quercetin, cinnamaldehyde and hirudin, have been reported to protect DRG neurons against such effects (Shi et al., 2013; Yang et al., 2016; Shi et al., 2017; Liu et al., 2020). Based on these findings, we hypothesized that JMT could exhibit neuroprotective effects by modulating NLRP3 inflammasome activation and pyroptosis in the DRG of diabetic rats.

Here, we confirmed the neuroprotective benefits of JMT treatment through behavioral and morphological assessments, and investigated the expression of NLRP3 inflammasome components and GSDMD in streptozocin (STZ)-induced diabetic rats. Our results offer new insights into the therapeutic mechanism of JMT, laying the foundation for a clinical application of JMT therapy in DNP.

## Material and Methods

### Drug Preparation

JMT is composed of twelve botanical drugs which are as follows: Semen Cuscutae, Fructus Ligustri Lucidi, Herba Ecliptae, Herba *Prunella* Vulgaris, Semen Litchi, Scorpio, Ramulus Cinnamoml, Rhizoma Corydalis, Semen Persicae, Senmen Cassiae, Radix et Rhizoma Asari, and Hirudo at a fixed ratio of 10: 10: 10: 10: 30: 3: 10: 10: 10: 30: 3: 3 (w/w), respectively (Sun et al., 2019). These botanical drugs were purchased from Beijing Tong Ren Tang Pharm Co., Ltd (Beijing, China) and authenticated by Prof. Xiaochun Liang according to the Chinese Pharmacopoeia (2015 Edition). Detailed information and voucher numbers for the botanical drugs are presented in Supplementary Table S1. The voucher specimens (No. JMT-17A-JMT-17L) were submitted to the Department of Traditional Chinese Medicine, Peking Union Medical College Hospital, Beijing, China. Alpha-lipoic acid (ALA) was purchased from Shandong Qidu Pharmaceutical Co., Ltd. (1.45mmol/tablet; Zibo City, Shandong, China, Lot No. H20100152) and was used as a positive control.

### Chemical Analysis of JMT

50 mg of JMT powder was extracted in the mixture of 400 μL methanol and 100 μL water. Ultra-high performance liquid chromatography/mass spectrometry (UPLC/MS) analysis was performed on a 1290 UPLC system (Agilent Technologies, Santa Clara, CA, United States) with an ACQUITY UPLC C18 column (Waters Corp, Milford, MA). The flow rate was 0.4 ml/min and the sample injection volume was 5 μL. The mobile phase consisted of 0.1% formic acid in water (A) and 0.1% formic acid in acetonitrile (B). The linear elution gradient program was as follows: 0–3.5 min, 95–85% A; 3.5–6 min, 85–70% A; 6–6.5, 70–70% A; 6.5–12 min, 70–30% A; 12–12.5 min, 30–30% A; 12.5–18 min, 30–0% A; 18–25 min, 0–0% A; 25–26 min, 0–95% A; 26–30 min, 95–95% A. The MS data was acquired by Q Exactive Focus mass spectrometer (Thermo Fisher Scientific, Waltham, MA, United States) with a mass range of 100–1500 m/z.

### Animals and Drug Treatment

Male Sprague-Dawley rats weighing 180–200 g were acquired from Vital River Laboratory Animal Technology (Beijing, China). The diabetic model was induced by a single intraperitoneal injection of streptozotocin (0.21 mmol/kg in citrate buffer; Sigma-Aldrich, St Louis, MO, United States), while control rats received a vehicle control of citrate buffer in equal volume. Blood glucose levels were measured 72 h after STZ administration using the glucometer (Abbott Molecular, Abbott Park, IL, United States). Rats with blood glucose levels ≥16.7 mmol/L were considered diabetic, and control rats with blood glucose levels <7.0 mmol/L were classified as normal control rats.

The rats were randomly divided into four groups (*n* = 8) as follows: 1) Con (normal control rats that only received distilled water); 2) DM (diabetic rats that also received distilled water); 3) DM + JMT (diabetic rats that received 0.88 g/kg/d JMT, 10-fold the dosage for a human adult); 4) DM + ALA (diabetic rats that received 0.48 mmol/kg/d ALA). We chose the JMT dosage which showed the best neuroprotective effects in STZ-induced diabetic rats based on our previous studies (Song et al., 2019; Song et al., 2021). ALA dosage chosen was based on published reports outlining STZ-induced diabetic rat models (Lee et al., 2018; Sadeghiyan et al., 2019). JMT powder and ALA tablets were suspended in distilled water, mixed thoroughly, and gavaged once daily for 12 weeks. Body weight and blood glucose levels were monitored throughout the treatment period.

After the treatment period ended, rats were anesthetized with sodium pentobarbital (0.24 mmol/kg, i.p.). Blood samples were collected from the carotid arteries, and were centrifuged at 4,000 r/min (10 min) for serum preparation. The serum was stored at -80 °C. The left L4-L6 DRGs were removed and rapidly frozen at -80 °C for molecular detection techniques, and the right side was harvested and fixed in 10% phosphate-buffered formalin for histological examination. The experiments were approved by the Institutional Animal Care and Use Committee of Peking Union Medical College Hospital (No. XHDW-2017–005), and all procedures were performed in accordance with the Principles of Laboratory Animal Care (NIH).

### Pain Behavior Test

After 12 weeks of treatment, mechanical withdrawal threshold (MWT) in response to mechanical stimuli was used to assess mechanical allodynia using Von Frey filaments (IITC Life Science, Woodland Hills, CA, United States) as described previously (Sun et al., 2019). Rats were individually placed in a clear plastic cage with mesh at the bottom. After a 15-min acclimation period, the hind paw was vertically stimulated by an electronic Von Frey probe, and the force was recorded in grams when the paw was withdrawn. Tail-flick latency (TFL) in response to heat-water was used to evaluate thermal hyperalgesia. The rats were wrapped in a towel and placed on the apparatus. The distal 5 cm of their tails were immersed in a hot-water bath (50 ± 0.5°C) and the tail withdrawal reflex was recorded in seconds (cut-off time 12 s) using a stopwatch (Yadlapalli et al., 2016). These tests were performed at 10 a.m. with three repeat measures for each rat within a 5-min interval.

### Enzyme-Linked Immunosorbent Assay (ELISA)

Serum IL-1β levels were measured, using a commercially available ELISA kit (eBioscience, San Diego, CA, United States), according to the manufacturer’s instructions. Optical density at 450 nm was measured with a plate reader, and sample values were calculated from standard curve analysis.

### Histological Analysis

The DRG tissues were processed, embedded in paraffin, and cut into 4 μm sections for histological analysis. After dewaxing and hydration, sections were stained with hematoxylin and 25% eosin for hematoxylin and eosin (H&E) staining. For Nissl’s staining, sections were stained with toluidine blue solution (Solarbio, Beijing, China) for 30 min at 50°C, followed by 95% ethanol to remove excess staining. Images were scanned and exported using a NanoZoomer Digital Slide Scanner (Hamamatsu Photonics, Hamamatsu, Shizuoka, Japan) at ×400 magnification.

### Immunohistochemistry

Following dewaxing and hydration, the sections were treated with hot citric acid buffer (pH 6.0) to repair antigens, incubated in 3% H_2_O_2_ and blocked with 10% goat serum. The sections were then incubated with the following primary antibodies: NLRP3 and IL-1β at 4°C overnight, followed by the appropriate HRP-conjugated secondary antibodies (Zhongshan Golden Bridge Biotechnology, Beijing, China). Detailed information on the antibodies is shown in Supplementary Table S2. After DAB staining, the sections were counterstained with hematoxylin, cleared in xylene, and mounted. Images were captured using a NanoZoomer Digital Slide Scanner (Hamamatsu Photonics, Hamamatsu, Shizuoka, Japan) at ×400 magnification. Quantitative analysis was carried out with NIH ImageJ software.

### Quantitative Real-Time RT-PCR

Total RNA was extracted from DRG tissues using Tissue RNA Purification Kit Plus (ES Science, Shanghai, China) according to the manufacturer’s protocol, and total RNA concentrations were tested using a spectrophotometer (Thermo Fisher Scientific, Waltham, MA, United States). Reverse transcription of the RNA (≤1 μg) was performed in a final volume of 20 μL (Absin Bioscience Inc., Shanghai, China). qRT-PCR was performed in duplicate in a Roche LightCycler 480 real-time PCR detection system using SYBR Green (Absin Bioscience Inc., Shanghai, China). The program was run with the following conditions: 2 min at 95°C, followed by 40 cycles of 15 s at 95°C, 25 s at 60°C and 30 s at 72°C. Relative mRNA levels were determined using the 2^−△△CT^ method. Results were expressed as relative mRNA expression in the treated groups compared to the normal control group. The primers for NLRP3, ASC, caspase-1, IL-1β, GSDMD and β-actin were synthesized by TSINGKE Biological Technology (Beijing, China), and the primer sequences are displayed in Supplementary Table S3.

### Western Blot Analysis

Total proteins from L4-L6 DRG were extracted with RIPA lysis buffer and determined using a BCA protein assay kit (Beyotime, Shanghai, China). Equal sized 30 μg proteins were separated in 10% SDS-PAGE (Beyotime, Shanghai, China) and transferred to PVDF membranes (Merck Millipore, Billerica, MA, United States). After blocking with 5% fat-free milk, the membranes were incubated with following primary antibodies: NLRP3, caspase-1, IL-1β, GSDMD and β-actin overnight at 4°C. Detailed information on the antibodies is presented in Supplementary Table S2. The corresponding secondary antibodies were probed after washing the membranes. Final results were visualized using an ECL kit (Merck Millipore, Billerica, MA, United States). The relative protein levels were analyzed with NIH ImageJ software.

### Statistical Analysis

GraphPad Prism 7.0 software (La Jolla, CA, United States) was used for statistical analysis. Data were presented as means ± standard error of the mean (SEM). The results between multiple groups were compared using one-way analysis of variance (ANOVA) followed by Tukey’s post hoc test. The correlations between two variables were assessed using Pearson correlation analysis. *p* < 0.05 was considered statistically significant.

## Results

### JMT did Not Affect Body Weight or Blood Glucose Levels in Diabetic Rats

**FIGURE 1:**
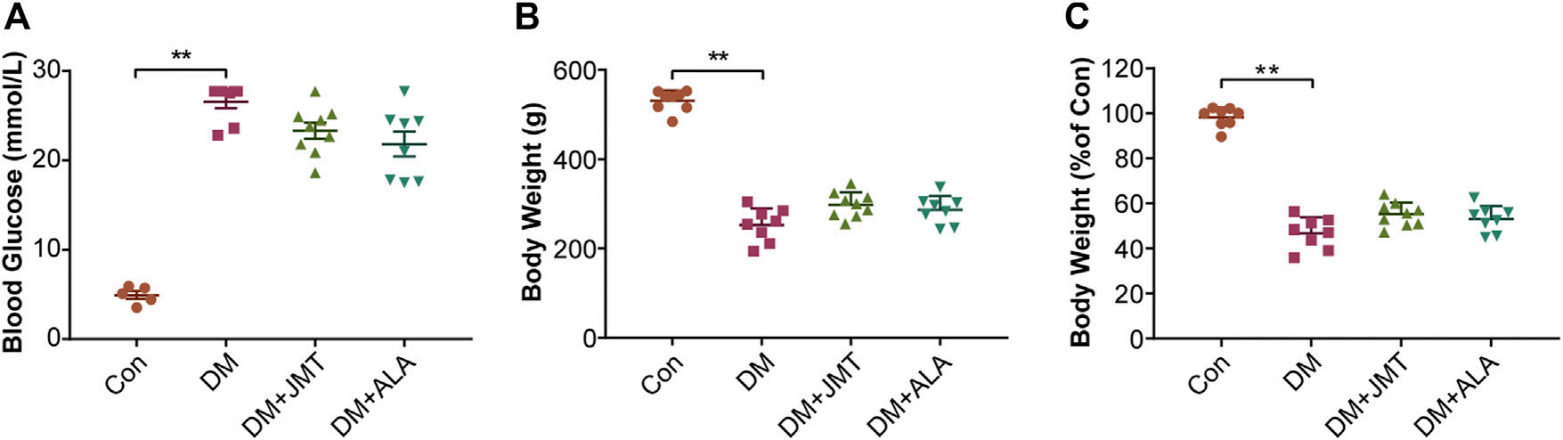
Effects of JMT on blood glucose levels and body weights in diabetic rats **(A)** Blood glucose **(B)** body weight and **(C)** body weight levels relative to the normal control group were measured at 12 weeks after STZ. The data are presented as mean ± SEM (n = 8 per group). ^**^ *p* < 0.01 *vs* Con group. Con, control; DM, diabetes mellitus; ALA, alpha-lipoic acid.

At 12 weeks after STZ injection, blood glucose levels were significantly increased in diabetic rats (Figure 1A), and body weights decreased by about 50% in diabetic rats compared with the Con group (Figures 1B,C). While JMT and ALA treatment had hypoglycemic and weight gaining trends, there was no significant difference in either body weights or blood glucose levels between the DM group and the JMT or ALA-treated DM groups (*p >* 0.05).

### JMT Improved DNP Phenotypes in Diabetic Rats

**FIGURE 2:**
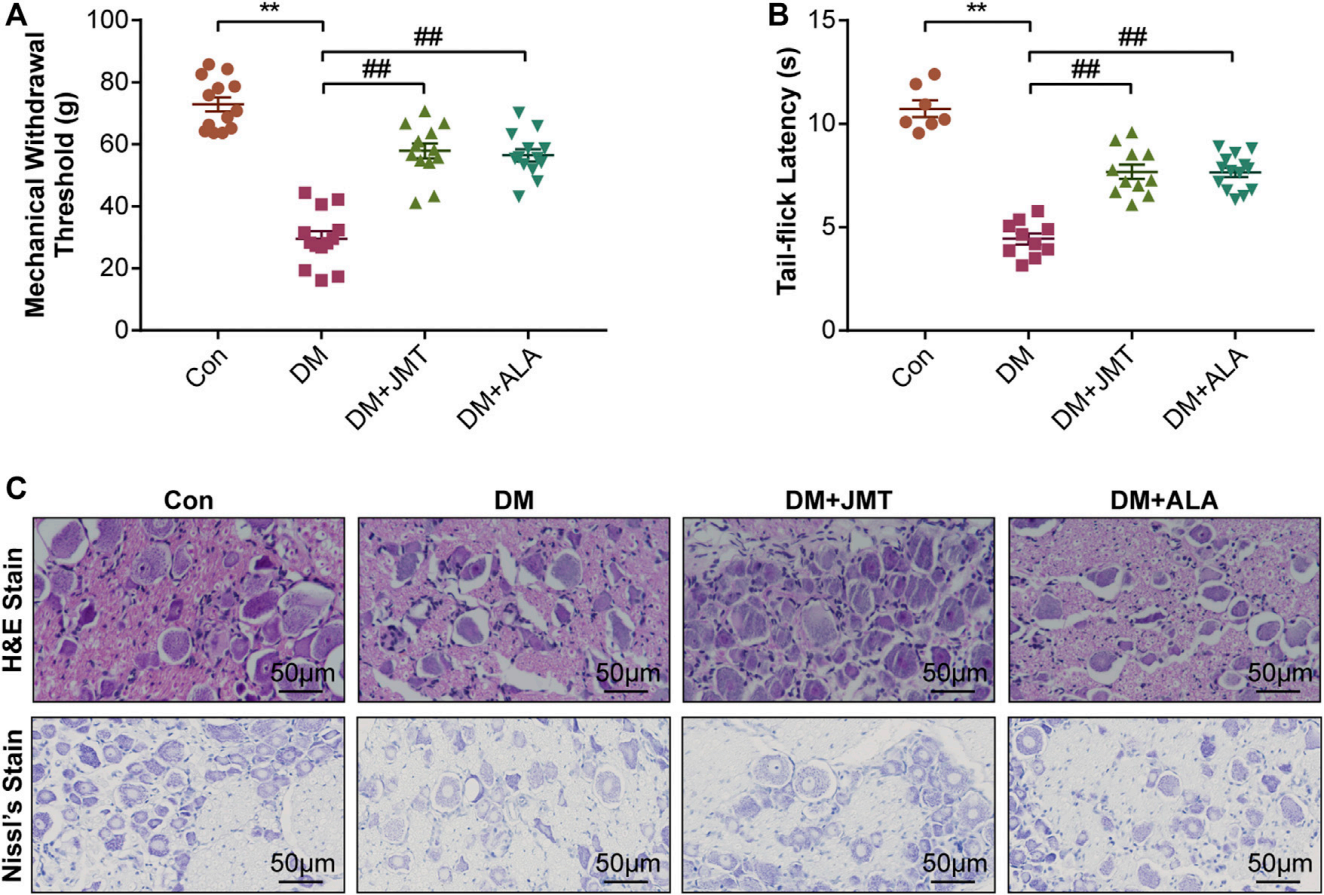
Effects of JMT on DNP phenotypes in diabetic rats **(A)** Mechanical withdrawal threshold (MWT) and tail-flick latency (TFL) levels **(B)** were assessed after 12 weeks of treatment. Representative images of **(C)** hematoxylin and eosin (H&E) and Nissl’s staining of Dorsal root ganglia (DRG) in different groups (magnification, ×400; scale bars: 50 μm). The data are presented as mean ± SEM (n = 8 per group). ^**^ *p* < 0.01 *vs* Con group, ^##^ *p* < 0.01 *vs* DM group. Con, control; DM, diabetes mellitus; ALA, alpha-lipoic acid.

MWT and TFL levels were used to evaluate the effects of JMT on mechanical allodynia and thermal hyperalgesia in diabetic rats. Compared to normal rats, diabetic rats had decreased MWT and TFL levels at 12 weeks after STZ injection. While JMT and ALA treatment partly reversed diabetes-induced decreases in MWT and TFL, there was no significant difference between the JMT and ALA groups (*p >* 0.05) (Figures 2A,B). In a word, both JMT and ALA alleviated mechanical allodynia and thermal hyperalgesia in diabetic rats, with ALA showing no predominance over JMT.

H&E and Nissl’s staining was used to assess the effects of JMT on DRG morphological injury in diabetic rats. According to H&E staining (Figure 2C), the cells were divided into two groups based on size, appearance, and histochemical reactions. Type A cells were larger and their cytoplasm occupied the entire cell, creating a light background. The nuclei of these neurons were generally lightly stained, with one large and less intensely stained central nucleolus. In contrast, type B cells had darker, more heterogeneously stained cytoplasm than type A. Type B cells were also usually smaller and had more evident vacuolation. Their nuclei were characterized by more basophilic and smaller chromatin condensations (Palanivelu et al., 2018). We found that type B cells were more abundant in the DM group compared to the Con group. Both the JMT and ALT-treated DM groups showed a reduction in Type B cells and an increase in type A cells with a dramatic reduction in vacuolation. Type A cells appeared to be more prevalent in the JMT group than the ALA group.

According to Nissl’s staining (Figure 2C), DRG neurons in the Con group showed clear structures with numerous Nissl’s bodies in the cytoplasm and nucleoli. The nuclei were large and round, and the nucleoli were in the middle and clearly visible. The Nissl bodies in the DRG of diabetic rats broke down and gradually disappeared, and even loss of neurons with vacuolar-like degeneration, the nuclei shifted and the nucleoli blurred, suggesting peripheral nerve damage. The morphological damage and Nissl’s body loss in DRG neurons improved in the JMT and ALA-treated DM groups. However, JMT showed no better effect on the neurological morphology of DRG than ALA.

### JMT Down-Regulated NLRP3 Inflammasome Activation in DRG of Diabetic Rats

**FIGURE 3:**
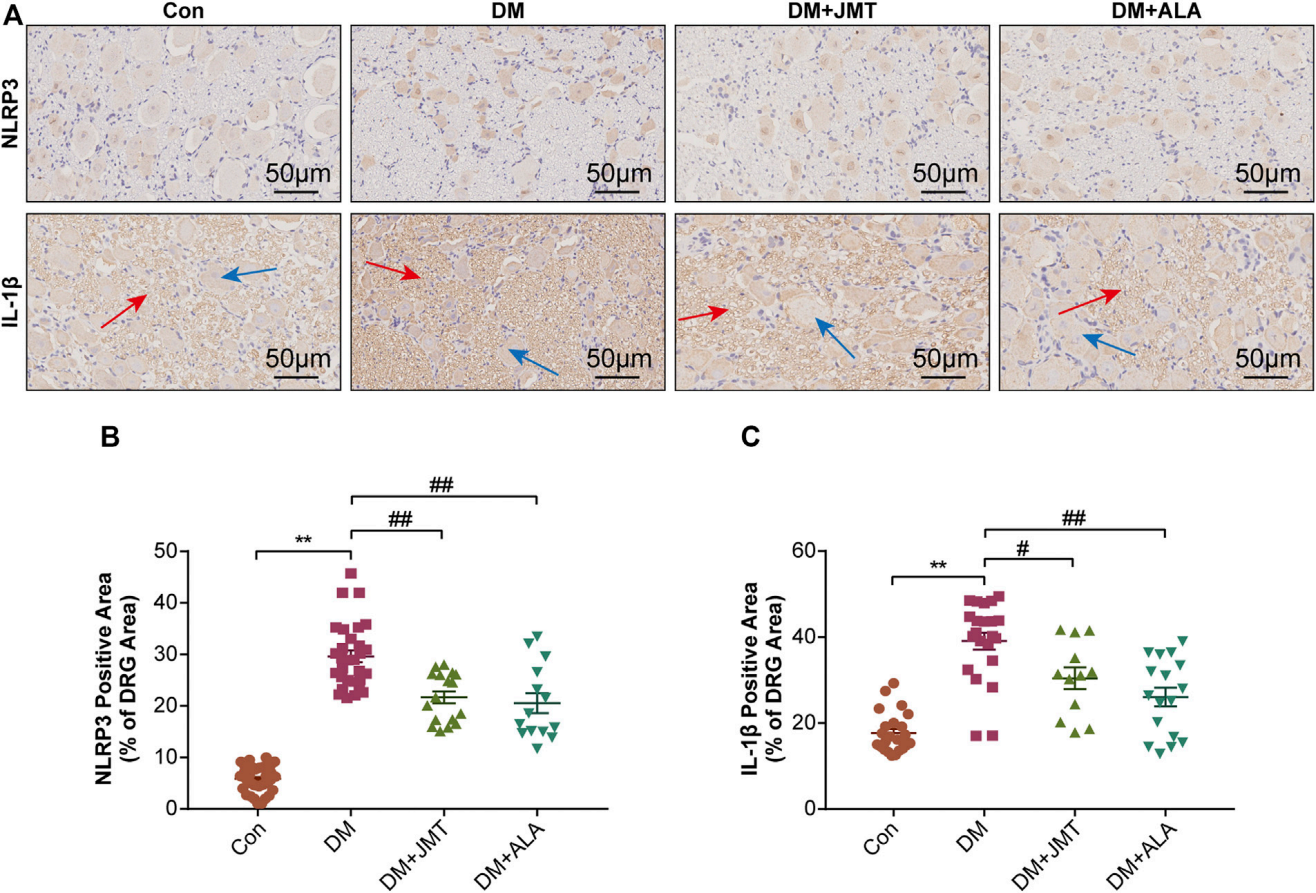
Immunohistochemistry sections of DRG tissues in rats **(A)** Representative immunohistochemical images of NLRP3 and IL-1β in different groups (magnification, ×400; scale bars: 50 μm). Quantitative analysis using immunohistochemistry images of **(B)** NLRP3 and **(C)** IL-1β positive-stained area relative to total tissue area. The data are presented as mean ± SEM (n = 6 per group). ^**^ *p* < 0.01 *vs* Con group, ^#^ *p* < 0.05 *vs* DM group, ^##^ *p* < 0.01 *vs* DM group. Con, control; DM, diabetes mellitus; ALA, alpha-lipoic acid.

**FIGURE 4:**
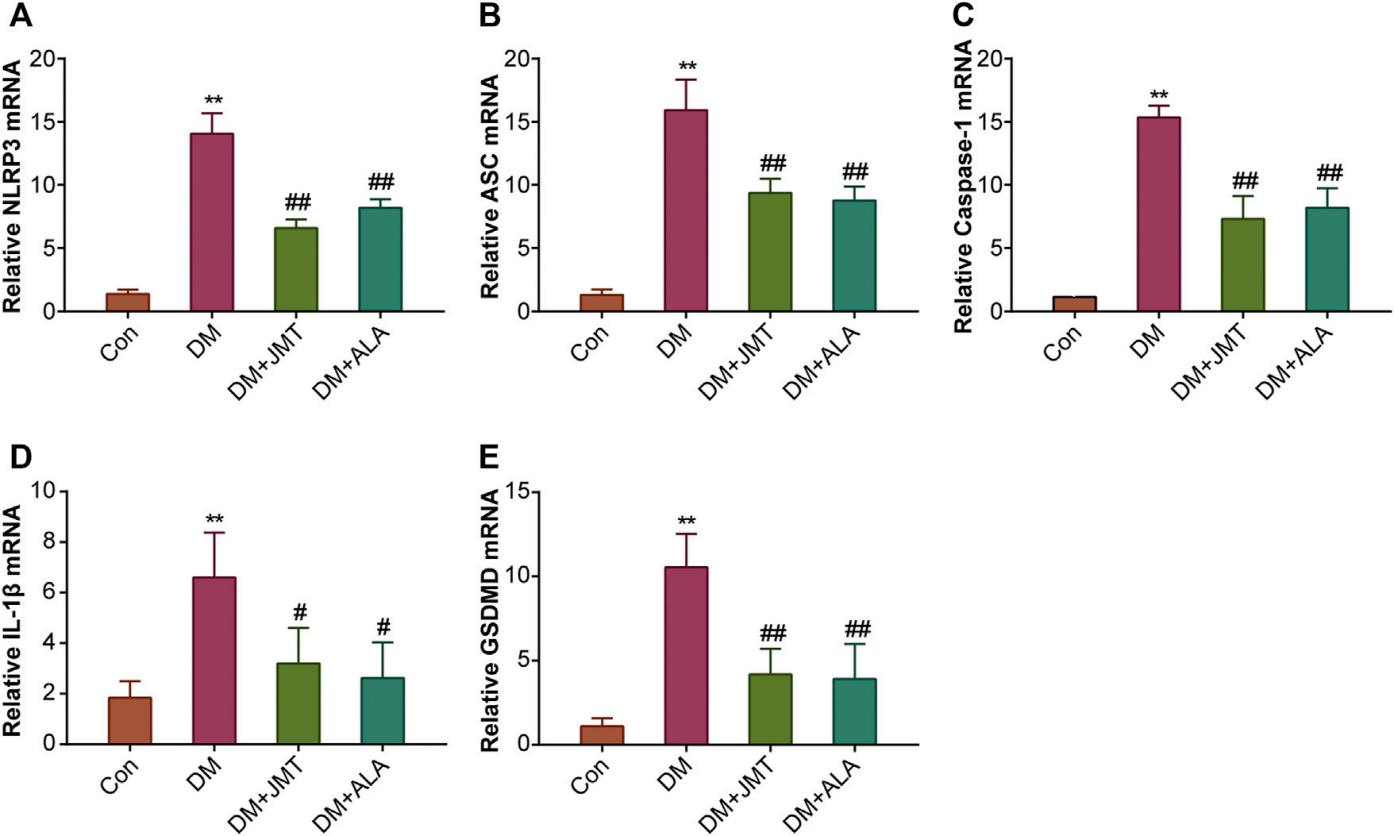
Effects of JMT on the mRNA levels of NLRP3 inflammasome components and GSDMD in DRG of diabetic rats **(A)** NLRP3 **(B)** ASC **(C)** caspase-1 **(D)** IL-1β and **(E)** GSDMD mRNA levels were measured by qPT-PCR analysis. Results were expressed as relative mRNA levels of target genes compared with the Con group. The data are presented as mean ± SEM (n = 3 per group). ^**^ *p* < 0.01 *vs* Con group, ^#^ *p* < 0.05 *vs* DM group, ^##^ *p* < 0.01 *vs* DM group. Con, control; DM, diabetes mellitus; ALA, alpha-lipoic acid.

**FIGURE 5:**
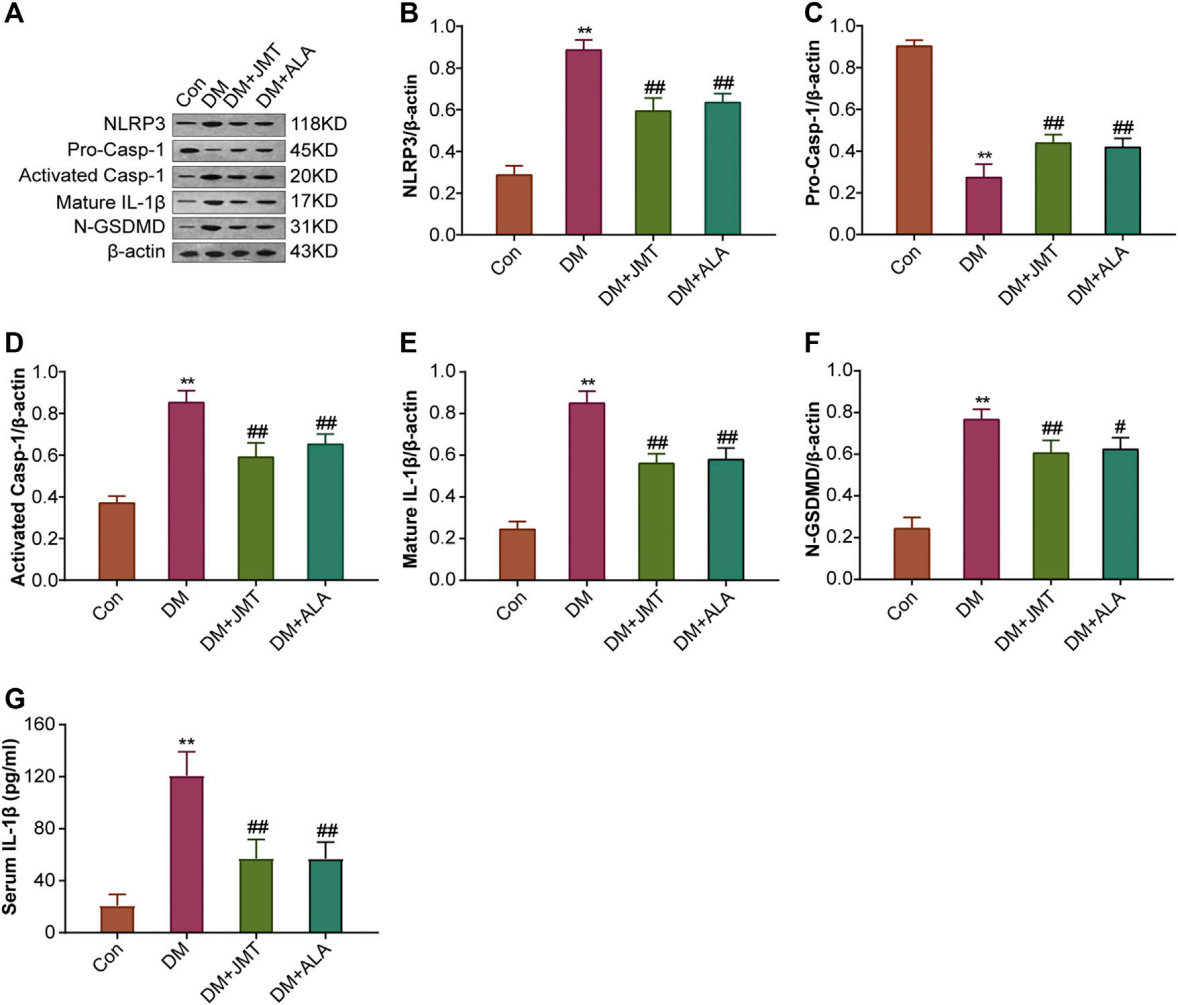
Effects of JMT on the protein expressions of NLRP3 inflammasome components and GSDMD in DRG of diabetic rats **(A)** Representative western blot images of NLRP3, pro-caspase-1, activated-caspase-1, IL-1β and GSDMD in DRG after 12 weeks. Quantitative analysis of **(B)** NLRP3 **(C)** pro-caspase-1 **(D)** activated-caspase-1 **(E)** IL-1β and **(F)** GSDMD protein expressions normalized to β-actin. The data are presented as mean ± SEM (n = 3 per group) **(G)** Serum IL-1β levels were analyzed using ELISA (n = 6 per group). ^**^ *p* < 0.01 *vs* Con group, ^#^ *p* < 0.05 *vs* DM group, ^##^ *p* < 0.01 *vs* DM group. Con, control; DM, diabetes mellitus; ALA, alpha-lipoic acid.

NLRP3 inflammasome consists of the receptor protein NLRP3, the adaptor protein apoptosis-associated speck-like protein (ASC), and the effector protein caspase-1, which response to caspase-1 activation and IL-1β maturation (Elliott and Sutterwala, 2015). We determined the cellular distribution of NLRP3 in DRG with immunohistochemistry (IHC). We found that NLRP3 was primarily distributed in the cytoplasm of type B DRG neurons, with increased expression in the DM group compared to the Con group. JMT treatment markedly attenuated NLRP3 expression in rats with diabetes, with no significant difference between the JMT and ALA-treated DM groups (*p >* 0.05) (Figures 3A,B). These findings were consistent with our qRT-PCR and western blot data (Figures 4A, 5A–B, ). We found elevated ASC and caspase-1 mRNA via qRT-PCR, and increased activated caspase-1 protein levels and lower pro-caspase-1 levels in diabetic rats compared to normal rats, indicating that NLRP3 inflammasome was activated and caspase-1 was cleaved in the DNP animal model. These changes were evidently restored by both JMT and ALA treatment, with no differences between the two drug groups (*p >* 0.05) (Figures 4B,C, 5A, C–D). Together, these data suggest that JMT can reduce NLRP3 inflammasome activation in DRG tissues under chronic hyperglycemic conditions.

### JMT Reduced GSDMD and IL-1β Expression in DRG of Diabetic Rats

GSDMD fragments were cleaved by activated caspase-1, allowing them to form membrane pores, which resulted in pyroptosis and IL-1β secretion and ultimately triggered an inflammatory cascade (Shi et al., 2015; Liu et al., 2016). Given this, we checked GSDMD and IL-1β expression in our animal model. IHC results showed that IL-1β was mainly distributed in the cytoplasm of DRG neurons and in satellite glial cells (SGCs), with increased expression in SGCs than in DRG (Figures 3A,C). Quantitative analysis showed that IL-1β and GSDMD mRNA and protein levels were increased in diabetic rats compared to normal control rats, and that JMT and ALA treatment partly normalized the mRNA and protein levels. However, there was no evident difference between the JMT and ALA groups (*p >* 0.05) (Figures 4D,E, 5A, 5E,F). Moreover, the maturation and secretion of IL-1β displayed a similar trend compared with GSDMD (Figure 5G).

**FIGURE 6:**
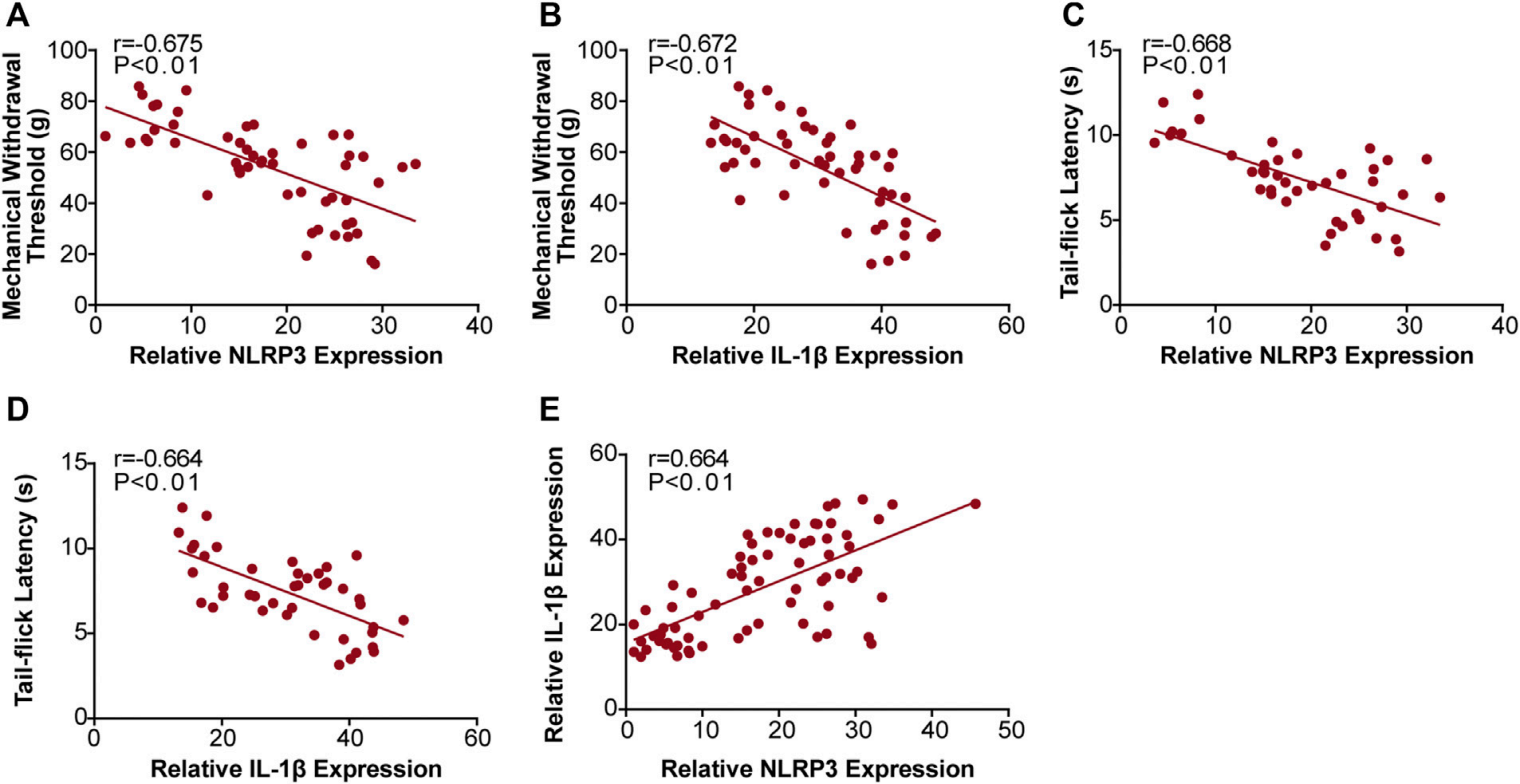
Correlation analysis between the behavioral phenotypes and NLRP3, IL-1β expression in diabetic rats. Analysis of the correlations between MWT levels and NLRP3, IL-1β expression **(A)** r (NLRP3/MWT) = −0.675 and **(B)** r (IL-1β/MWT) = −0.672. Correlation analysis between TFL levels and NLRP3, IL-1β expression **(C)** r (NLRP3/TFL) = −0.668 and **(D)** r (IL-1β/TFL) = −0.664. Analysis of the correlations between NLRP3 and IL-1β expression **(E)** r (NLRP3/IL-1β) = 0.664. r: correlation coefficient.

We found that MWT levels were negatively correlated with NLRP3 (r = −0.675, Figure 6A), and IL-1β (r = −0.672, Figure 6B) expression, and that TFL levels were also negatively correlated with NLRP3 (r = −0.668, Figure 6C), and IL-1β (r = −0.664, Figure 6D) expression. Moreover, we found that NLRP3 expression positively correlated with IL-1β (r = 0.664, Figure 6E) expression. We confirmed these results with qRT-PCR and western bolt analysis, indicating that JMT improved DNP development by decreasing the NLRP3/IL-1β pathway.

## Discussion

Our study demonstrated that JMT treatment could relieve DNP by improving behavioral damage such as mechanical allodynia and thermal hyperalgesia. We also found JMT to improve morphological injury in the DRG of diabetic rats. These effects were negatively associated with NLRP3 inflammasome-induced inflammation and pyroptosis.

We used a single intraperitoneal STZ-injection to induce a type 1 diabetic rat model, which manifested with hyperglycemia and weight loss (Furman, 2015). Neither JMT nor ALA affected blood glucose and body weight levels at 12 weeks after STZ injection. Our previous study also showed that no significant differences in body weight and blood glucose levels were found between the DM and JMT-treated DM groups at 16 weeks (Yin et al., 2015). In a STZ-induced diabetic rat model, ALA did not affect fasting glucose levels, random glucose levels, or Hemoglobin (Hb) A1c levels at 12 and 24 weeks (Lee et al., 2018). However, we found that both JMT and ALA improved abnormal glucose metabolism in patients with type 2 diabetes (Liang et al., 1999; Karkabounas et al., 2018). Our animal model was a type 1 diabetic model induced by STZ, in which pancreatic β-cells are destroyed, making it difficult to reduce blood glucose levels.

After 12 weeks, diabetic rats performed more nociceptive behaviors and had impaired nerve structures compared with normal control rats, which were defined as DNP phenotypes (Biessels et al., 2014). Twelve weeks of JMT administration improved not only nociceptive behaviors, including declined MWT and TFL, but also ameliorated morphological damage of the DRG, suggesting that JMT is an effective therapy for DNP in diabetic rats. The presence of basophilic Nissl bodies in the cytoplasm is a hallmark feature of a normal neuron. Nissl bodies synthesize proteins, including the structural proteins required for organelles production and the enzymes needed for neurotransmitter and neuromodulator synthesis. Thus, Nissl bodies are treated as a morphological indicator of functional neuronal activity. Compared with the routine pathological method of H&E staining, Nissl’s staining uses basic dyes (such as toluidine blue, cresyl violet, methylene blue and thionine, etc.) which can clearly visualize the structure of Nissl bodies (Kiernan et al., 1998). Recently, Nissl’s staining has been applied to assessing the morphological damage of DRG tissues (Zhang et al., 2016; Perez et al., 2018). Toluidine blue staining results showed reduced Nissl body formation and cytoplasmic vacuolization in the DRG of diabetic rats, which were partly rescued by JMT treatment, consistent with our previous finding (Liu et al., 2013). These morphological changes suggest that DRG neuronal damage could be caused by the chronic hyperglycemic conditions used in our experiment.

Pyroptosis, a form of programmed necrosis characterized by cell lysis and a release of cellular contents, is mediated by inflammatory caspases (Jorgensen and Miao, 2015). In the context of innate immune effector mechanisms, pyroptosis has long been regarded as caspase-1-mediated monocyte death (Bergsbaken et al., 2009). Caspase-1 is activated through canonical inflammasome sensors like NLRP3 upon detection of microbial components and endogenous dangers (Broz and Dixit, 2016). Caspase-4/5/11 directly binds to cytoplasmic lipopolysaccharide, which expands the potential spectrum of pyroptosis mediators and indicates that pyroptosis is not cell type-specific (Shi et al., 2014). Both caspase-1 and caspase-4/5/11 cleave GSDMD, a gasdermin-family member, releasing its gasdermin-N domain to perforate the plasma membrane and induce cell swelling and osmotic lysis (Shi et al., 2015; Ding et al., 2016; Liu et al., 2016). The role of NLRP3 inflammasome-mediated pyroptosis in diabetic neuropathy has recently attracted increasing attention. NLRP3 inflammasome activation and IL-1β release have been observed in the DRG of mice with high-fat diet induced prediabetic neuropathy and palmitate-treated DRG neurons (Xu et al., 2019). Consistent with the above study, we found elevated NLRP3 levels in the DRG neurons of type 2 diabetic mice and rats with painful neuropathy (Thakur et al., 2020). High glucose-stimulated Schwann cells sustained NLRP3 inflammasome (NLRP3, ASC, caspase-1) activation, IL-1β and IL-18 maturation and GSDMD cleavage (Cheng et al., 2020). We found that JMT treatment attenuated presently elevated mRNA and protein levels of NLRP3 and its essential constituents ASC and caspase-1. We also observed GSDMD release and IL-1β expression in the DRG tissues, and IL-1β secretion in the serum of JMT-treated DNP rats. These effects were not superior compared to the positive control ALA.

JMT is a prescription based on the basic theory of TCM (QU et al., 2008). Semen Cuscutae, Fructus Ligustri Lucidi and Herba Ecliptae tonify *qi* and *yin* of the kidney; Hirudo and Scorpion promote blood circulation to remove blood stasis; Ramulus Cinnamoml and Herba Asarum warm and activate channels and collaterals; Rhizoma Corydalis promote *qi* circulation to relieve pain; Herba *Prunella* vulgaris and Semen Litchi soften and resolve hard lumps; Semen Persicae and Semen Cassiae relieve constipation. According to the pharmacology of Chinese materia medica, these botanical drugs have anti-oxidative, anti-inflammatory, anti-diabetic, and neuroprotective pharmacological effects.

Among the 261 chemical compounds we identified by UPLC/MS analysis, the major compounds of JMT were flavonoids, terpenoids and phenols (Supplementary Table S4 **,** Supplementary Figure S1–2). Quercetin, hesperidin and luteolin have been reported to attenuate nociceptive behavior and increase NCV in STZ-induced rats with diabetic neuropathy. These observations are likely caused by activation of the Nrf2/HO-1 pathway, inhibition of phosphorylated p38 mitogen-activated protein kinase (p38MAPK), and Wnt/β-catenin pathway signaling (Visnagri et al., 2014; Li et al., 2015; Yang et al., 2019). Apigenin alleviated peripheral neuropathy including axonal degradation, myelin fragmentation, *trans*-dedifferentiation, and Schwann cell proliferation by scavenging free radicals (Kim et al., 2019). The antioxidant and antiapoptotic properties of naringenin prevented retinal neurodegeneration in diabetic retinopathy (Al-Dosari et al., 2017). Recently, eriodictyol was found to protect retinal ganglial cells against high glucose-induced oxidative stress, inflammation, and neuronal apoptosis via Nrf2/HO-1 pathway activation (Lv et al., 2019). Kaempferol has been reported to have anti-oxidative, anti-inflammatory, neuroprotective, and anti-microbial effects (Calderon-Montano et al., 2011). All these compounds have antidiabetic activities (Al-Ishaq et al., 2019; Bai et al., 2019). Aside from these known compounds, the pharmaceutical effects of JMT on DNP treatment likely rely on the integrated utility of multiple compounds.

However, our study has some limitations. We did not measure pyroptosis directly by evaluating lactate dehydrogenase (LDH) release or propidium iodide influx in high glucose-induced DRG neurons, and future research is needed using *in vitro* models. Also, the composition of JMT is complex, so we discussed the pharmaceutical effects of some of the individual compounds. The mechanism of interaction between the different compounds requires further study. Finally, The dose used in this study was comparatively high which is associated with the risk of artefacts. Therefore, it is desirable to assess this preparation further at a more pharmacologically relevant dose range.

## Conclusion

**FIGURE 7:**
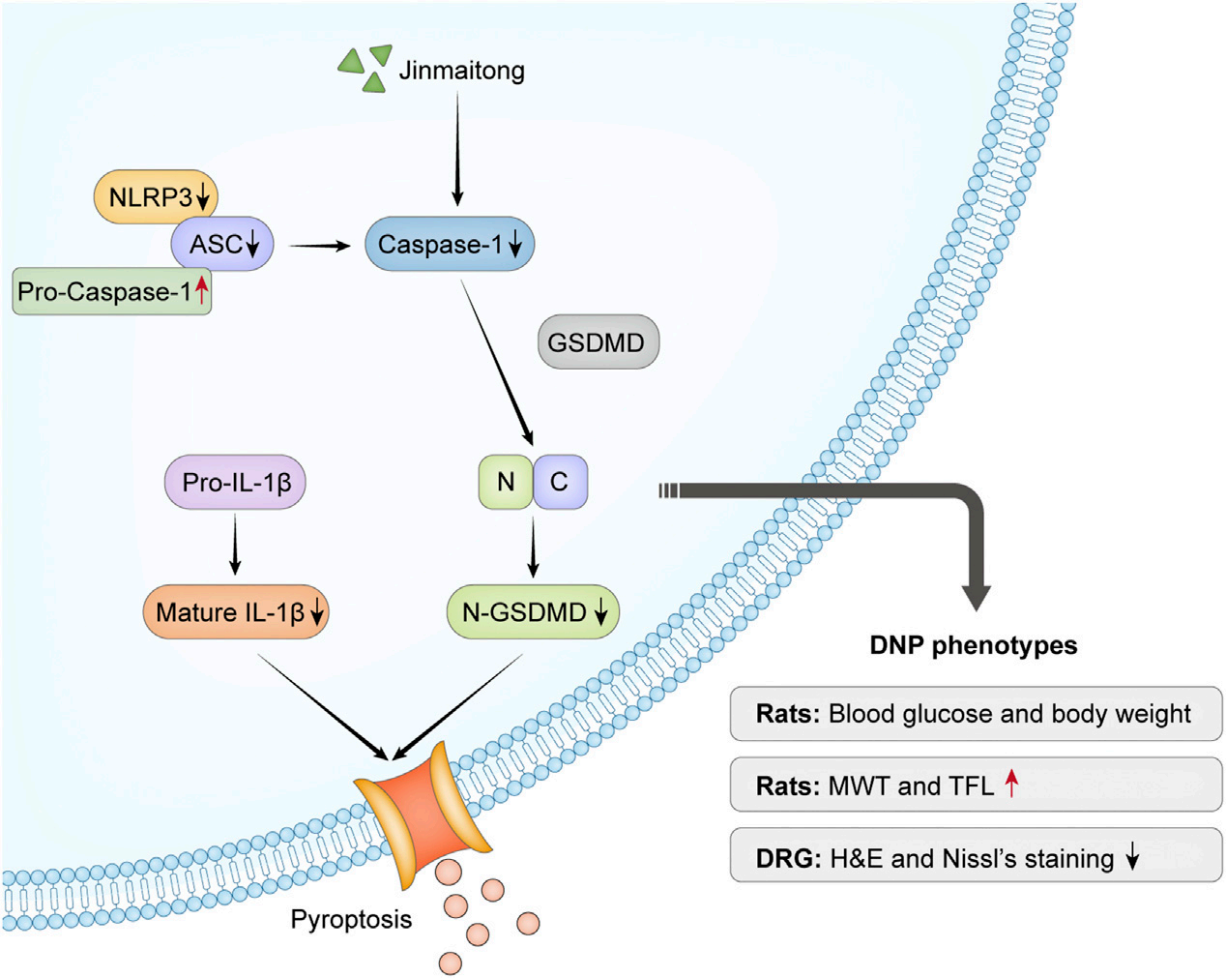
Overview mechanism of neuroprotective effects of JMT on DNP. Chronic hyperglycemia stimulated NLRP3 inflammasome activation, which led to nociceptive behavior and morphological injury in diabetic rats. The activation of caspase-1 induced by NLRP3 inflammasome promoted IL-1β secretion and GSDMD cleavage. JMT effectively relieved DNP through the downregulation of NLRP3 inflammasome activation and pyroptosis. Red arrows indicate up-regulation by JMT, black arrows show down-regulation by JMT.

Our study confirmed that JMT alleviated the behavioral and morphological damage of DNP by reducing NLRP3 inflammasome and pyroptosis, independent of a hypoglycemic effect. (Figure 7). These results provide insight into the potential therapeutic mechanisms of JMT, providing new evidence for using JMT as an alternative treatment strategy for DNP. Future research is needed to identify novel active compounds in JMT and evaluate their mechanism of action.

## Data Availability

The original contributions presented in the study are included in the article/Supplementary Material, further inquiries can be directed to the corresponding authors.
