# Supplementary material for: Jinmaitong Alleviates Diabetic Neuropathic Pain Through Modulation of NLRP3 Inflammasome and Gasdermin D in Dorsal Root Ganglia of Diabetic Rats
Source: Front Pharmacol. 2021 Nov 3;12:679188. doi: 10.3389/fphar.2021.679188 (PMC8596020; doi:10.3389/fphar.2021.679188)
Supplement: Supplementary file 1 [file DataSheet1.PDF]

Supplementary Material

Supplementary Table 1. The crude drug materials in Jinmaitong (JMT).

Voucher number	Drug name	Biological origin	Chinese name	Ratio (w/w)
JMT-17A	Semen Cuscutae	the seeds of *Cuscuta chinensis* Lam.	Tu-Si-Zi	10
JMT-17B	Fructus Ligustri Lucidi	the seeds of *Ligustrum lucidum* Ait.	Nv-Zhen-Zi	10
JMT-17C	Herba Ecliptae	the herb of *Eclipta prostrata* L.	Mo-Han-Lian	10
JMT-17D	Herba Prunella vulgaris	the herb of *Prunella vulgaris* L.	Xia-Ku-Cao	10
JMT-17E	Semen Litchi	the seeds of *Litchi chinensis* Sonn.	Li-Zhi-He	30
JMT-17F	Scorpio	the whole body of *Buthus martensii* K.	Quan-Xie	3
JMT-17G	Ramulus Cinnamoml	the tender stem of *Cinnamomum cassia* Presl.	Gui-Zhi	10
JMT-17H	Rhizoma Corydalis	the rhizoma of *Corydalis yanhusuo* W. T. Wang	Yan-Hu-Suo	10
JMT-17I	Semen Persicae	the seeds of *Prunus persica* L.	Tao-Ren	10
JMT-17J	Semen Cassiae	the seeds of *Cassia obtusifolia* L.	Jue-Ming-Zi	30
JMT-17K	Radix et Rhizoma Asari	the radix and rhizoma of *Asarum heterotropiodes* F.	Xi-Xin	3
JMT-17L	Hirudo	*Hirudo nipponica* W	Shui-Zhi	3

Supplementary Table 2. Detailed information of the primary antibodies.

Name of antibody	Manufacture (Host)	Working dilution	Applications
NLRP3	Abcam ((Rabbit)	1:200	IHC
IL-1β	R&D (Goat)	1:150	IHC
NLRP3	Abcam (Rabbit)	1:1000	WB
Caspase-1	Abcam (Rabbit)	1:1000	WB
IL-1β	Santa Cruz (Mouse)	1:1000	WB
GSDMD	Santa Cruz (Mouse)	1:500	WB
β-actin	Santa Cruz (Mouse)	1:1000	WB

Note: IHC, immunohistochemistry; WB, western blot.

Supplementary Table 3. Primer sequences for qRT-PCR.

Gene		Primer sequence
NLRP3	Forward	5′-CCAGAGCCTCACTGAACTGG-3′
	Reverse	5′-AGCATTGATGGGTCAGTCCG-3′
ASC	Forward	5′-GGACAGTACCAGGCAGTTCG-3′
	Reverse	5′-GTCACCAAGTAGGGCTGTGT-3′
Caspase-1	Forward	5′-CTGGACTGCGGTATTGAGAC-3′
	Reverse	5′-CCGGGTGCGGTAGAGTAAGC-3′
IL-1β	Forward	5′-CGTGCGTGACATTAAAGAG-3′
	Reverse	5′-TTGCCGATAGTGATGACC-3′
GSDMD	Forward	5′-TGAATGTGTACTCGCTGAGTGTGG-3′
	Reverse	5′-CAGCTGCTGCAGGACTTTGTG-3′
β-actin	Forward	5′-CCCATCTATGAGGGTTACGC-3′
	Reverse	5′-TTTAATGTCACGCACGATTTC-3′

Supplementary Table 4. The ingredients separation and identification of JMT by UPLC/MS analysis

No.	t_R (min)	Name	Formula	Class	$[MH]^-$ (m/z)	Peak Area
1	0.60	L(+)-Arginine	C6H14N4O2	Amino acid derivatives	173.1048	2.28E+07
2	0.62	MANNITOL	C6H14O6	Organooxygen compounds	181.0723	1.65E+08
3	0.67	D-Gluconic acid	C6H12O7	Organic acids and derivatives	195.0514	1.45E+09
4	0.67	Inositol	C6H12O6	Organic oxygen compounds	179.0564	4.66E+08
5	0.67	Xylose	C5H10O5	Carbohydrates and derivatives	149.0458	7.72E+07
6	0.67	Citric acid	C6H8O7	Carboxylic acids and derivatives	191.0203	2.96E+09
7	0.69	Fumaric acid	C4H4O4	Organic acids and derivatives	115.0040	1.58E+08
8	0.72	Sucrose	C12H22O11	Carbohydrates and derivatives	341.1101	2.27E+08
9	0.75	D-(+)-Malic acid	C4H6O5	Hydroxy acids and derivatives	133.0146	1.59E+09
10	0.88	Uridine	C9H12N2O6	Alkaloids	243.0625	2.23E+07
11	0.88	5-OXO-D-PROLINE	C5H7NO3	Amino acid derivatives	128.0356	1.30E+08
12	0.88	Aconitic Acid	C6H6O6	Organic acids and derivatives	173.0096	1.61E+08
13	0.89	N-Acetyl-DL-glutamic acid	C7H11NO5	Amino acid derivatives	188.0570	5.08E+07
14	0.89	Atranol	C8H8O3	Miscellaneous	151.0403	3.43E+07
15	0.89	3,4-Dihydroxyphenylglycol	C8H10O4	Phenols	169.0510	1.38E+07
16	0.93	Citraconic acid	C5H6O4	Fatty Acyls	129.0197	9.12E+07
17	0.93	SUCCINATE	C4H6O4	Organic acids and derivatives	117.0197	6.62E+08
18	1.02	L-ISOLEUCINE	C6H13NO2	Amino acid derivatives	130.0878	1.63E+08
19	1.05	Gallic acid	C7H6O5	Phenols	169.0148	1.66E+08
20	1.07	Sorbose	C6H12O6	Benzene and substituted derivatives	179.0565	9.28E+07
21	1.07	adipic acid	C6H10O4	Fatty acids	145.0512	2.04E+07
22	1.21	2-METHYLMALEATE	C5H6O4	Fatty Acyls	129.0198	4.13E+07
23	1.35	Pyrogallol	C6H6O3	Phenols	125.0248	1.45E+07

24	1.41	kojic acid	C6H6O4	Organoheterocyclic compounds	141.0198	1.78E+07
25	1.46	2,3-DIHYDROXYBENZOATE	C7H6O4	Phenylpropanoids and polyketides	153.0197	3.38E+07
26	1.50	Phenylalanine	C9H11NO2	Amino acid derivatives	164.0722	1.07E+08
27	1.51	4-Methoxysalicylic acid	C8H8O4	Phenols	167.0355	1.24E+08
28	1.54	Caffeic acid	C9H8O4	Phenylpropanoids	179.0354	1.85E+07
29	1.64	Glutaric acid	C5H8O4	Organic acids and derivatives	131.0354	5.42E+07
30	1.65	Calcium pantothenate	C9H17NO5.1/2 Ca	Alkaloids	218.1040	8.31E+07
31	1.74	Coumarin + 1O + 1MeO, O-Hex-Hex	C22H28O14	Phenylpropanoids	515.1424	6.13E+08
32	1.79	Catechol	C6H6O2	Phenols	109.0297	1.21E+08
33	1.84	4-Methoxyphenylacetic acid	C9H10O3	Phenolic acids	165.0561	2.85E+07
34	1.90	3,4-Dihydroxyphenylethanol	C8H10O3	Phenols	153.0562	6.58E+08
35	1.90	4-Methylcatechol	C7H8O2	Phenols	123.0454	1.60E+08
36	1.90	Methyl-4-hydroxy-3-methoxybenzoate	C9H10O4	Benzoic acids and derivatives	181.0510	3.36E+07
37	1.99	Harpagide	C15H24O10	Iridoids	363.1307	1.79E+07
38	2.01	Orsellinic acid	C8H8O4	Phenols	167.0355	1.68E+07
39	2.07	Orcinol gentiobioside	C19H28O12	Phenols	447.1517	1.11E+07
40	2.11	Lamiide	C17H26O12	Iridoids	421.1364	3.48E+07
41	2.16	P-Anisic acid	C8H8O3	Phenols	151.0402	2.79E+07
42	2.19	Shanzhiside	C16H24O11	Iridoids	391.1250	1.97E+07
43	2.23	4-Hydroxyphenyllactic acid	C9H10O4	Phenylpropanoids	181.0509	1.34E+08
44	2.26	Loganic acid	C16H24O10	Iridoids	375.1308	9.04E+07
45	2.30	Swertiamarin	C16H22O10	Iridoids	419.1210	3.17E+08
46	2.30	Piperonylic Acid	C8H6O4	Organic acids and derivatives	165.0195	6.60E+07
47	2.31	Carpachromene	C20H16O5	Flavonoids	335.0909	5.63E+08
48	2.38	L-Tryptophan	C11H12N2O2	Organoheterocyclic compounds	203.0829	3.71E+07

49	2.42	[(2R,3S,4S,5R,6R)-6-[(2S,3S,4S,5R)-3,4-dihydroxy-2,5-bis(hydroxymethyl)oxolan-2-yl]oxy-3,4,5-trihydroxyoxan-2-yl]methyl 4-hydroxybenzoate	C19H26O13	Phenylpropanoids and polyketides	461.1305	2.23E+07
50	2.43	Ajugol	C15H24O9	Terpenoids	393.1409	6.35E+07
51	2.45	Protocatechualdehyde	C7H6O3	Phenols	137.0246	5.38E+08
52	2.51	Fraxetin	C10H8O5	Coumarins and derivatives	207.0299	2.58E+07
53	2.59	p-Hydroxy-cinnamic acid	C9H8O3	Phenylpropanoids	163.0400	1.02E+08
54	2.59	(1R,3R,4S,5R)-1,3,4-trihydroxy-5-[(E)-3-(4-hydroxyphenyl)prop-2-enoyl]oxycyclohexane-1-carboxylic acid	C16H18O8	Phenylpropanoids	337.0937	2.28E+09
55	2.61	Forsythoside E	C20H30O12	Phenylpropanoids	461.1676	1.12E+07
56	2.76	Chlorogenic acid	C16H18O9	Phenylpropanoids	353.0883	8.88E+08
57	2.76	Quinic acid	C7H12O6	Organooxygen compounds	191.0565	2.04E+08
58	2.90	Benzyl alcohol + Hex-Hex	C19H28O11	Organooxygen compounds	477.1627	1.10E+09
59	2.90	osmanthuside H	C19H28O11	Flavonoids	431.1570	1.84E+08
60	2.94	4-Hydroxyphenylacetic acid	C8H8O3	Phenols	151.0402	2.77E+07
61	2.96	Glutamylphenylalanine	C14H18N2O5	Carboxylic acids and derivatives	293.1242	2.05E+08
62	3.04	Cryptochlorogenic acid	C16H18O9	Phenylpropanoids	353.0883	3.21E+08
63	3.05	methyl chlorogenate	C17H20O9	Phenylpropanoids	367.1037	5.18E+06
64	3.07	Coumaroyl Hexoside	C15H18O8	Phenylpropanoids	325.0935	8.97E+07
65	3.08	6,7-Dihydroxycoumarin	C9H6O4	Phenylpropanoids	177.0199	3.57E+08
66	3.13	2',4'-DIHYDROXYACETOPHENONE	C8H8O3	Benzene and substituted derivatives	151.0403	6.60E+07
67	3.29	Amygdalin	C20H27NO11	Phenols	502.1568	1.71E+10
68	3.41	geniposide	C17H24O10	Iridoids	387.1301	2.82E+07
69	3.44	Gentisic acid	C7H6O4	Xanthones	153.0197	5.02E+07
70	3.47	4-hydroxybenzaldehyde	C7H6O2	Phenols	121.0299	2.06E+08
71	3.56	Syringic Acid	C9H10O5	Phenols	197.0461	1.23E+07

72	3.70	7,8-Dihydroxycoumarin	C9H6O4	Phenylpropanoids	177.0197	2.27E+07
73	3.72	Secoxyloganin	C17H24O11	Iridoids	403.1258	1.89E+08
74	3.79	sweroside	C16H22O9	Terpenoids	357.1196	2.46E+07
75	3.79	Shikimic acid	C7H10O5	Phenolic acids	173.0458	2.18E+08
76	3.83	5,7-dihydroxy-2-(4-hydroxyphenyl)-6,8-bis[3,4,5-trihydroxy-6-(hydroxymethyl)oxan-2-yl]chromen-4-one	C27H30O15	Flavonoids	593.1522	5.22E+07
77	3.85	Benzyl alcohol + Hex-Pen	C18H26O10	Miscellaneous	401.1458	6.69E+07
78	4.03	Demethylwedelolactone	C15H8O7	Phenylpropanoids	299.0205	1.18E+08
79	4.03	Delphinidin	C15H11O7	Tetraterpenes	301.0362	2.93E+08
80	4.07	Prulaurasin	C14H17NO6	Terpenoids	294.0987	4.55E+07
81	4.29	methyl (1S)-7-hydroxy-7-methyl-1-[(2S,3R,4S,5S,6R)-3,4,5-trihydroxy-6-(hydroxymethyl)oxan-2-yl]oxy-4a,5,6,7a-tetrahydro-1H-cyclopenta[c]pyran-4-carboxylate	C17H26O10	Terpenoids	389.1462	1.28E+07
82	4.41	Gossypetin-8-C-glucoside	C21H20O13	Flavonoids	479.0842	3.05E+08
83	4.41	(-)-12-hydroxyjasmonic acid	C12H18O4	Jasmonic acid	225.1136	9.71E+08
84	4.43	Isoleucine	C6H13NO2	Amino acid derivatives	130.0875	8.14E+06
85	4.47	Luteolin-4'-O-glucoside	C21H20O11	Flavonoids	447.0934	7.44E+07
86	4.54	Coumaroyl quinic acid	C16H18O8	Phenylpropanoids	337.0936	3.01E+07
87	4.59	vanillic acid	C8H8O4	Organic acids and derivatives	167.0355	1.36E+07
88	4.62	Shanzhiside methyl ester	C17H26O11	Terpenoids	405.1407	4.21E+07
89	4.65	Aloenin	C19H22O10	Phenols	409.1178	2.61E+07
90	4.74	3-Phenyllactic acid	C9H10O3	Carbonyl	165.0561	1.31E+08
91	4.80	Methyl gallate	C8H8O5	Phenols	183.0301	1.21E+08
92	4.86	methyl (1S,4aR,7aR)-4a-hydroxy-7-(hydroxymethyl)-1-[(2S,3R,4S,5S,6R)-3,4,5-trihydroxy-6-(hydroxymethyl)oxan-2-yl]oxy-5,7a-dihydro-1H-cyclopenta[c]pyran-4-carboxylate	C17H24O11	Miscellaneous	403.1258	5.14E+07

93	4.86	Suberic acid	C8H14O4	Fatty acids	173.0824	3.58E+07
94	4.87	Isoscopoletin	C10H8O4	Coumarins and derivatives	191.0354	1.16E+07
95	4.96	Ferulic acid	C10H10O4	Phenylpropanoids	193.0512	1.50E+07
96	4.99	1-[[(2S,3R,11bR)-3-ethyl-9,10-dimethoxy-2,3,4,6,7,11b-hexahydro-1H-benzo[a]quinolizin-2-yl]methyl]-7-methoxy-3,4-dihydro-2H-isoquinolin-6-one	C28H36N2O4	Alkaloids	463.2555	2.58E+08
97	5.13	(2S,3S)-2-(3,4-dihydroxyphenyl)-3,7-dihydroxy-2,3-dihydrochromen-4-one	C15H12O6	Flavonoids	287.0566	2.59E+07
98	5.14	Taxifolin	C15H12O7	Flavonoids	303.0515	1.90E+07
99	5.16	Procyanidin B2	C30H26O12	Procyanidins	577.1365	2.10E+07
100	5.17	Quercetin-3-O-galactoside	C21H20O12	Flavonoids	463.0899	7.98E+08
101	5.24	Procyanidin A2	C30H24O12	Procyanidins	575.1199	2.97E+08
102	5.25	5,7-dihydroxy-2-(4-hydroxyphenyl)-3-[(2S,3R,4S,5S,6R)-3,4,5-trihydroxy-6-[[(2S,3R,4S,5S)-3,4,5-trihydroxyoxan-2-yl]oxymethyl]oxan-2-yl]oxychromen-4-one	C26H28O15	Flavonoids	579.1369	1.98E+07
103	5.34	(2Z)-2-[4-[2-[2-(3,4-dihydroxyphenyl)ethoxy]-2-oxoethyl]-5-methoxycarbonyl-2-[(2S,3R,4S,5S,6R)-3,4,5-trihydroxy-6-(hydroxymethyl)oxan-2-yl]oxy-4H-pyran-3-ylidene]acetic acid	C25H30O15	Phenols	569.1531	2.25E+08
104	5.45	[(2R,3R,4S,5R,6R)-6-[2-(3,4-dihydroxyphenyl)ethoxy]-3,5-dihydroxy-4-[(2R,3R,4R,5R,6S)-3,4,5-trihydroxy-6-methyloxan-2-yl]oxyoxan-2-yl]methyl (E)-3-(3,4-dihydroxyphenyl)prop-2-enoate	C29H36O15	Phenylpropanoids	623.2002	2.44E+08
105	5.45	3-(2-HYDROXYPHENYL)PROPANOATE	C9H10O3	Phenylpropanoids and polyketides	165.0561	2.78E+07
106	5.58	Hexose + C13H17O3	C19H28O9	Miscellaneous	399.1676	1.60E+07
107	5.63	1,3-Dicaffeoylquinic acid	C25H24O12	Phenylpropanoids	515.1203	9.96E+08
108	5.69	Kaempferol-3-O-glucoside	C21H20O11	Flavonoids	447.0935	4.96E+08
109	5.72	Isoliquiritin	C21H22O9	Chalcones	417.1203	2.24E+07

110	5.80	[(1aS,1bS,2S,5aR,6S,6aS)-1a-(hydroxymethyl)-2-[(2S,3R,4S,5S,6R)-3,4,5-trihydroxy-6-(hydroxymethyl)oxan-2-yl]oxy-2,5a,6,6a-tetrahydro-1bH-oxireno[5,6]cyclopenta[1,3-c]pyran-6-yl] benzoate	C22H26O11	Miscellaneous	465.1404	6.11E+07
111	5.83	(2S,3R,4S)-4-(2-{[(2E)-3-(3,4-Dihydroxyphenyl)-2-propenoyl]oxy}ethyl)-2-(beta-D-glucopyranosyloxy)-3-vinyl-3,4-dihydro-2H-pyran-5-carboxylic acid	C25H30O13	Phenylpropanoids	537.1613	1.31E+07
112	5.85	Rhamnetin	C16H12O7	Flavonoids	315.0513	1.38E+09
113	5.88	Apigenin-7-O-glucoside	C21H20O10	Flavonoids	431.0997	4.58E+07
114	5.95	Dihydrokaempferol	C15H12O6	Flavonoids	287.0566	2.12E+07
115	6.03	4-Hydroxybenzoic acid	C7H6O3	Phenols	137.0246	2.87E+07
116	6.04	Naringin	C27H32O14	Flavonoids	579.1729	1.81E+07
117	6.06	Azelaic acid	C9H16O4	Fatty Acyls	187.0977	1.72E+08
118	6.08	Danshensu	C9H10O5	Phenylpropanoids	197.0453	2.33E+07
119	6.08	Umbelliferone	C9H6O3	Phenylpropanoids	161.0244	1.09E+08
120	6.08	Rosmarinic acid	C18H16O8	Phenylpropanoids	359.0781	2.86E+09
121	6.11	7-hydroxy-2-(4-hydroxyphenyl)-5-[(2S,3R,4S,5S,6R)-3,4,5-trihydroxy-6-(hydroxymethyl)oxan-2-yl]oxy-2,3-dihydrochromen-4-one	C21H22O10	Flavonoids	433.1144	2.32E+07
122	6.19	columbianetin	C14H14O4	Phenylpropanoids	245.0818	4.80E+07
123	6.19	Eurycomanone	C20H24O9	Terpenoids	407.1356	1.81E+08
124	6.23	Phloridzin	C21H24O10	Flavonoids	435.1302	6.03E+07
125	6.26	Genistin	C21H20O10	Flavonoids	431.0996	7.44E+07
126	6.34	Homoplantaginin	C22H22O11	Flavonoids	461.1093	2.77E+08
127	6.38	Daidzein	C15H10O4	Flavonoids	253.0502	3.10E+07
128	6.45	Specneuzhenide	C31H42O17	Terpenoids	685.2384	1.73E+08

129	6.46	5,7-dihydroxy-2-(4-hydroxyphenyl)-6-[(2S,3R,4R,5S,6R)-3,4,5-trihydroxy-6-(hydroxymethyl)oxan-2-yl]-8-[(2S,3R,4R,5R,6S)-3,4,5-trihydroxy-6-methyloxan-2-yl]chromen-4-one	C27H30O14	Flavonoids	577.1591	5.14E+08
130	6.53	Cinnamic acid	C9H8O2	Phenylpropanoids	193.0512	1.98E+07
131	6.56	Petunidin-3-O-beta-glucopyranoside	C22H23O12	Flavonoids	477.1040	1.55E+08
132	6.56	rhodioloside	C14H20O7	Phenols	299.1140	1.36E+07
133	6.61	Flavonol base + 3O, O-Hex	C21H20O11	Flavonoids	447.0936	6.40E+07
134	6.65	Kaempferol-3-O-glucoside-7-O-rhamnoside	C27H30O15	Flavonoids	593.1522	1.77E+07
135	6.68	(+)-Usniacin	C18H16O7	Organic acids and derivatives	343.0828	1.08E+08
136	6.75	Scutellarein	C15H10O6	Flavonoids	285.0411	2.98E+07
137	6.80	Kaempferol 3-O-arabinoside	C20H18O10	Flavonoids	417.0839	2.79E+07
138	6.88	Atractyloside A	C21H36O10	Terpenoids	493.2306	3.41E+07
139	6.93	Abscisic acid	C15H20O4	Sesquiterpenoids	263.1292	8.70E+07
140	6.94	Quercetin	C15H10O7	Flavonoids	301.0362	1.73E+08
141	7.04	Sebacic acid	C10H18O4	Fatty Acyls	201.1136	2.86E+07
142	7.15	Wedelolactone	C16H10O7	Phenylpropanoids	313.0355	2.04E+09
143	7.22	Tectoridin	C22H22O11	Flavonoids	461.1094	7.80E+08
144	7.40	Pelargonidin-3-O-glucoside	C21H21O10	Flavonoids	431.0996	3.19E+08
145	7.45	Chrysophanol 8-O-beta-D-glucoside	C21H20O9	Flavonoids	415.1039	3.68E+05
146	7.51	Morin	C15H10O7	Flavonoids	301.0362	8.66E+07
147	7.54	naringenin-7-O-glucoside	C21H22O10	Flavonoids	433.1143	2.83E+08
148	7.58	methyl (5Z)-5-ethylidene-4-[2-[[(2R,3S,4S,5R,6R)-5-[2-[(3Z)-3-ethylidene-5-methoxycarbonyl-2-[(2S,3R,4S,5S,6R)-3,4,5-trihydroxy-6-(hydroxymethyl)oxan-2-yl]oxy-4H-pyran-4-yl]acetyl]oxy-3,4-dihydroxy-6-[2-(4-hydroxyphenyl)ethoxy]oxan-2-yl]methoxy]-2-oxoethyl]-6-	C48H64O27	Miscellaneous	1071.3605	1.67E+07

		[(2S,3R,4S,5S,6R)-3,4,5-trihydroxy-6-(hydroxymethyl)oxan-2-yl]oxy-4H-pyran-3-carboxylate				
149	7.70	Biochanin-7-O-glucoside	C22H22O10	Flavonoids	491.1208	9.91E+08
150	7.72	5,7-dihydroxy-6-methoxy-2-[4-[(2S,3R,4S,5S,6R)-3,4,5-trihydroxy-6-(hydroxymethyl)oxan-2-yl]oxyphenyl]chromen-4-one	C22H22O11	Flavonoids	461.1095	1.93E+07
151	7.75	Naringenin	C15H12O5	Flavonoids	271.0618	7.81E+07
152	7.78	isosakuranetin-7-O-neohesperidoside	C28H34O14	Flavonoids	593.1896	1.18E+07
153	7.81	Complanatuside	C28H32O16	Flavonoids	623.1631	7.58E+06
154	7.91	Nodakenin	C20H24O9	Coumarins and derivatives	407.1355	1.86E+07
155	7.94	Herbacetin	C15H10O7	Flavonoids	301.0362	8.23E+06
156	8.02	Jasmonic acid	C12H18O3	Fatty acids	209.1185	3.80E+07
157	8.09	Flavone base + 3O, 1MeO	C16H12O6	Flavonoids	299.0569	3.87E+07
158	8.11	Usnic acid	C18H16O7	Phenols	343.0827	1.48E+07
159	8.14	Isorhamnetin	C16H12O7	Flavonoids	315.0514	3.67E+07
160	8.18	Salvianolic acid C	C26H20O10	Phenylpropanoids	491.1002	1.88E+07
161	8.26	Iridin	C24H26O13	Flavonoids	521.1317	8.88E+06
162	8.29	trifolirhizin	C22H22O10	Isoflavonoids	445.1141	1.31E+07
163	8.43	Ginsenoside Rh2 (S-FORM)	C36H62O8	Terpenoids	667.4450	1.53E+07
164	8.47	Aurantio-obtusin beta-D-glucoside	C23H24O12	Flavonoids	491.1206	1.52E+07
165	8.49	Baicalein	C15H10O5	Flavonoids	269.0459	1.59E+07
166	8.66	Methylophiopogonanone A	C19H18O6	Flavonoids	341.1039	1.36E+07
167	8.78	FA 18:1+3O	C18H34O5	Miscellaneous	329.2339	1.64E+09
168	8.80	Cianidanol	C15H14O6	Phenols	289.0725	8.67E+08
169	8.98	Glycitein	C16H12O5	Flavonoids	283.0617	5.99E+07
170	8.98	Diethyl-phthalate	C12H14O4	Benzoic acids and derivatives	221.0821	6.53E+06
171	8.98	cirsimarin	C23H24O11	Flavonoids	475.1260	6.61E+07

172	9.03	Amentoflavone	C30H18O10	Flavonoids	537.0814	5.25E+06
173	9.07	Goniothalenol	C13H12O4	Lignans	231.0666	9.33E+06
174	9.09	Eupafolin	C16H12O7	Flavonoids	315.0513	5.21E+08
175	9.13	Emodin	C15H10O5	Quinones	269.0460	7.43E+06
176	9.21	Brazilein	C16H12O5	Miscellaneous	283.0617	8.70E+06
177	9.21	(2S,3S,4S,5R,6R)-6-[[(3S,6aR,6bS,8aS,14bR)-4,4,6a,6b,11,11,14b-heptamethyl-8a-[(2S,3R,4S,5S,6R)-3,4,5-trihydroxy-6-(hydroxymethyl)oxan-2-yl]oxycarbonyl-1,2,3,4a,5,6,7,8,9,10,12,12a,14,14a-tetradecahydropicen-3-yl]oxy]-3,4,5-trihydroxyoxane-2-carboxylic acid	C42H66O14	Terpenoids	793.4426	6.52E+06
178	9.40	matairesinol	C20H22O6	Phenylpropanoids	357.1388	5.23E+06
179	9.45	alpha-Hederin	C41H66O12	Terpenoids	795.4552	5.49E+07
180	9.51	(2S,3S)-3,5,7-trihydroxy-2-(4-hydroxyphenyl)-8-(3-methylbut-2-enyl)-2,3-dihydrochromen-4-one	C20H20O6	Flavonoids	355.1200	4.32E+05
181	9.61	Inermin	C16H12O5	Flavonoids	283.0617	9.65E+08
182	9.61	Polygalaxanthone III	C25H28O15	Xanthones	567.1303	7.34E+06
183	9.79	Ginsenoside compound K	C36H62O8	Terpenoids	667.4452	8.10E+06
184	9.83	Pinocembrin	C15H12O4	Flavonoids	255.0667	5.62E+07
185	9.91	Echinocystic acid-3-O-glucoside	C36H58O9	Terpenoids	633.4045	9.54E+08
186	9.91	Hederagenin base + O-Hex	C36H58O9	Terpenoids	679.4112	1.69E+07
187	10.12	Fallacinol	C16H12O6	Quinones	299.0564	1.18E+07
188	10.18	6-Gingerol	C17H26O4	Phenols	293.1765	2.90E+07
189	10.20	Blinin	C22H32O6	Terpenoids	437.2211	7.04E+06
190	10.21	Atractylenolide III	C15H20O3	Sesquiterpenoids	247.1341	6.22E+06
191	10.22	Eupatilin	C18H16O7	Flavonoids	343.0828	7.80E+08
192	10.28	Medicagenic acid	C30H46O6	Triterpenoids	501.3228	8.59E+06
193	10.30	Jaceosidin	C17H14O7	Flavonoids	329.0678	1.33E+07

194	10.36	Genistein	C15H10O5	Flavonoids	269.0460	3.22E+07
195	10.53	[(2S,3R,4S,5S,6R)-3,4,5-trihydroxy-6-(hydroxymethyl)oxan-2-yl] (1R,2R,4aS,6aS,6bR,10R,11R,12aR)-1,10,11-trihydroxy-1,2,6a,6b,9,9,12a-heptamethyl-2,3,4,5,6,6a,7,8,8a,10,11,12,13,14b-tetradecahydropicene-4a-carboxylate	C36H58O10	Terpenoids	649.3984	5.83E+06
196	10.85	Pectolinarigenin	C17H14O6	Flavonoids	313.0718	2.85E+07
197	10.89	Isoimperatorin	C16H14O4	Coumarins and derivatives	269.0826	9.15E+06
198	10.95	[(2R)-2-[(E,2S,4R)-4,6-dimethyloct-6-en-2-yl]-6-oxo-2,3-dihydropyran-3-yl] (2E,4E,6S)-8-hydroxy-6-(hydroxymethyl)-4-methylocta-2,4-dienoate	C25H38O6	Miscellaneous	433.2597	5.27E+06
199	10.97	Aleuretic Acid	C16H32O5	Organic acids and derivatives	303.2178	1.02E+07
200	11.06	Hydroxygenkwanin	C16H12O6	Flavonoids	299.0571	3.85E+07
201	11.09	Momordin Ic	C41H64O13	Terpenoids	763.4330	3.78E+06
202	11.10	Iristectorigenin B	C17H14O7	Flavonoids	329.0678	5.84E+07
203	11.14	(1R,2R,4aS,6aS,6bR,10S,12aR,14bS)-1,8,10-trihydroxy-1,2,6a,6b,9,9,12a-heptamethyl-2,3,4,5,6,6a,7,8,8a,10,11,12,13,14b-tetradecahydropicene-4a-carboxylic acid	C30H48O5	Terpenoids	487.3443	3.17E+07
204	11.23	Spiculisporic acid	C17H28O6	Miscellaneous	327.1826	1.07E+07
205	11.30	13-HOTrE	C18H30O3	Organic acids and derivatives	293.2125	9.00E+06
206	11.37	Lauryl sulfate	C12H26O4S	Miscellaneous	265.1484	8.62E+07
207	11.50	DGMG 18:3	C33H56O14	Miscellaneous	675.3625	1.21E+07
208	11.62	Kaempferol	C15H10O6	Flavonoids	285.0405	7.31E+07
209	11.76	18alpha-glycyrrhetinic acid	C30H46O4	Terpenoids	469.3330	4.55E+06
210	11.80	Methyl hexadecanoate	C17H34O2	Fatty Acyls	315.2541	4.75E+08
211	11.82	(1S,4aR,6aS,6bR,9R,10R,11R,12aR,14bS)-1,10,11-trihydroxy-9-(hydroxymethyl)-2,2,6a,6b,9,12a-hexamethyl-1,3,4,5,6,6a,7,8,8a,10,11,12,13,14b-tetradecahydropicene-4a-carboxylic acid	C30H48O6	Terpenoids	503.3381	2.52E+07

212	11.89	Magnolol	C18H18O2	Lignans	265.1239	1.28E+07
213	11.92	Dihydrocapsaicin	C18H29NO3	Alkaloids	306.2077	7.30E+06
214	11.96	Di-n-butyl phthalate	C16H22O4	Organic acids and derivatives	277.1447	1.59E+07
215	12.01	2,4,6-trihydroxy-5-[1-(4-hydroxy-1,1,4,7-tetramethyl-1a,2,3,4a,5,6,7a,7b-octahydrocyclopropa[h]azulen-7-yl)-3-methylbutyl]benzene-1,3-dicarbaldehyde	C28H40O6	Terpenoids	453.2625	2.98E+07
216	12.15	Asiatic acid	C30H48O5	Terpenoids	533.3499	3.22E+07
217	12.35	12,13-EODE	C18H32O3	Fatty acids	295.2283	2.05E+09
218	12.60	FA 18:2+1O	C18H32O3	Miscellaneous	295.2283	4.92E+06
219	12.64	16-Hydroxyhexadecanoic acid	C16H32O3	Fatty Acyls	271.2282	1.01E+07
220	12.87	9(S)-HOTrE	C18H30O3	Fatty acids	293.2124	2.39E+08
221	12.96	FA 18:1+1O	C18H34O3	Miscellaneous	297.2436	2.89E+08
222	12.99	isosakuranetin	C16H14O5	Flavonoids	285.0776	1.07E+07
223	13.23	13-HODE	C18H32O3	Lipids	295.2283	4.20E+07
224	13.30	Linoleic acid	C18H32O2	Fatty Acyls	279.2327	3.68E+07
225	13.30	(5S,9R)-14-(hydroxymethyl)-5,9-dimethyltetracyclo$[11.2.1.0^{1,10}.0^{4,9}]$hexadecane-5-carboxylic acid	C20H32O3	Terpenoids	319.2288	7.18E+06
226	13.39	Corosolic acid	C30H48O4	Terpenoids	471.3479	1.51E+07
227	13.45	9,10-EODE	C18H32O3	Fatty acids	295.2284	2.25E+08
228	14.00	Palmitic Acid	C16H32O2	Fatty Acyls	255.2329	4.03E+07
229	14.15	3-O-Acetyl-16alpha-hydroxytrametenolic acid	C32H50O5	Terpenoids	513.3590	2.04E+08
230	14.15	Roccellic Acid	C17H32O4	Miscellaneous	299.2228	1.80E+07
231	14.16	Periplogenin	C23H34O5	Terpenoids	389.2336	6.33E+06
232	14.38	ISOPALMITIC ACID	C16H32O2	Fatty acids	255.2329	1.33E+07
233	14.56	Glabrolide	C30H44O4	Terpenoids	467.3170	4.69E+06
234	14.56	Pristimerin	C30H40O4	Terpenoids	463.2838	2.35E+08

235	14.61	Protolichesterinic acid	C19H32O4	Terpenoids	323.2228	7.04E+06
236	14.62	Abietic acid	C20H30O2	Terpenoids	301.2174	1.18E+08
237	14.64	(1R,2R,4S,7R,8S,12R)-7-(furan-3-yl)-1,8,12,17,17-pentamethyl-3,6,16-trioxapentacyclo[9.9.02,4.02,8.012,18]icos-13-ene-5,15,20-trione	C26H30O7	Terpenoids	453.1960	8.57E+06
238	14.75	Linolenic acid	C18H30O2	Fatty Acyls	277.2179	5.14E+07
239	14.79	LPC 16:0	C24H50NO7P	Lipids	540.3301	7.33E+07
240	14.92	Celastrol	C29H38O4	Terpenoids	449.2687	2.31E+07
241	15.03	2',4'-Dihydroxychalcone	C15H12O3	Flavonoids	239.0720	4.81E+06
242	15.24	Docosahexanoic acid	C22H32O2	Fatty acids	327.2331	5.18E+07
243	15.24	(2R,3R,4R,5R,6S)-2-[[(2R,3S,4S,5R,6S)-6-(4-ethenylphenoxy)-3,4,5-trihydroxyoxan-2-yl]methoxy]-6-methyloxane-3,4,5-triol	C20H28O10	Miscellaneous	427.1585	3.69E+06
244	15.25	Ecliptasaponin A	C36H58O9	Terpenoids	633.4032	8.63E+06
245	15.31	Isoxanthohumol	C21H22O5	Flavonoids	353.1433	2.17E+07
246	15.32	Palmitoleic acid	C16H30O2	Fatty acids	253.2176	7.57E+06
247	15.49	Arachidonic acid	C20H32O2	Fatty acids	303.2339	1.81E+08
248	15.55	5-Ethoxy-10-gingerol	C23H38O4	Phenols	377.2711	1.71E+07
249	15.56	Nicotinic acid	C6H5NO2	Alkaloids	122.0251	1.18E+07
250	15.61	Deoxycholic acid	C24H40O4	Terpenoids	391.2849	7.48E+06
251	16.06	9-HODE	C18H32O3	Lipids	295.2283	2.21E+07
252	16.14	Ginsenoside F1	C36H62O9	Terpenoids	637.4340	5.33E+07
253	16.24	Eicosenoic acid	C20H38O2	Lipids	309.2809	1.03E+08
254	16.31	Alisol A 24-acetate	C32H52O6	Terpenoids	577.3763	1.01E+07
255	16.45	alternariol monomethyl ether	C15H12O5	Phenylpropanoids	271.0619	7.77E+06
256	16.72	Oleic acid	C18H34O2	Fatty acids	281.2493	2.04E+08
257	16.72	Sorbitol	C6H14O6	Organic oxygen compounds	181.0721	5.52E+07
258	17.14	Ginkgolic Acid C15:1	C22H34O3	Phenolic acids	345.2445	4.26E+06

259	17.44	Maslinic acid	C30H48O4	Terpenoids	471.3480	4.31E+06
260	17.77	Fucoxanthin	C42H58O6	Terpenoids	657.4137	4.75E+06
261	26.44	3-[(Carboxycarbonyl)amino]-L-alanine	C5H8N2O5	Alkaloids	174.9563	7.62E+08

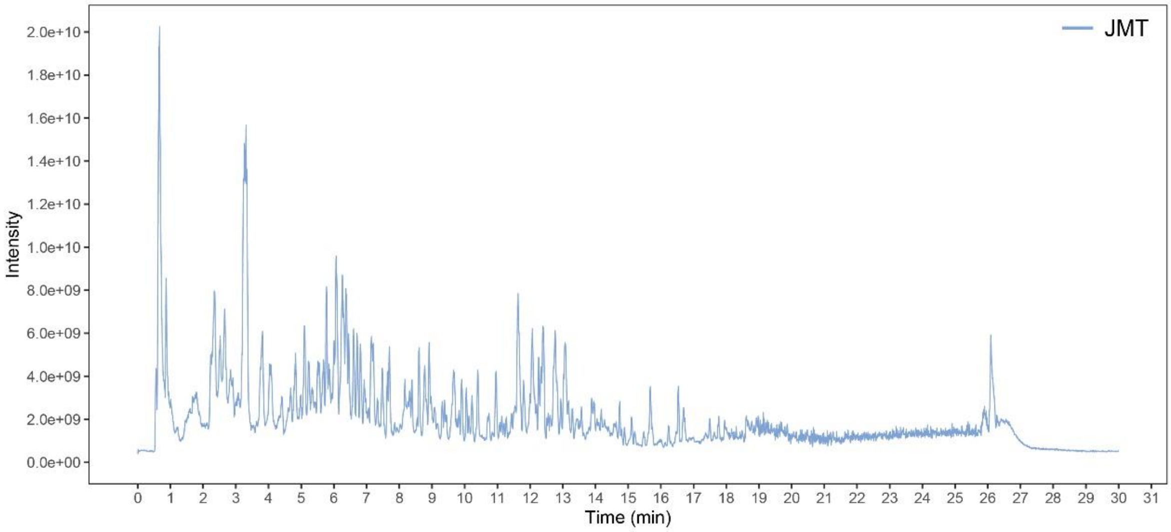

Supplementary Figure 1. The UPLC chromatogram of JMT in negative ion mode.

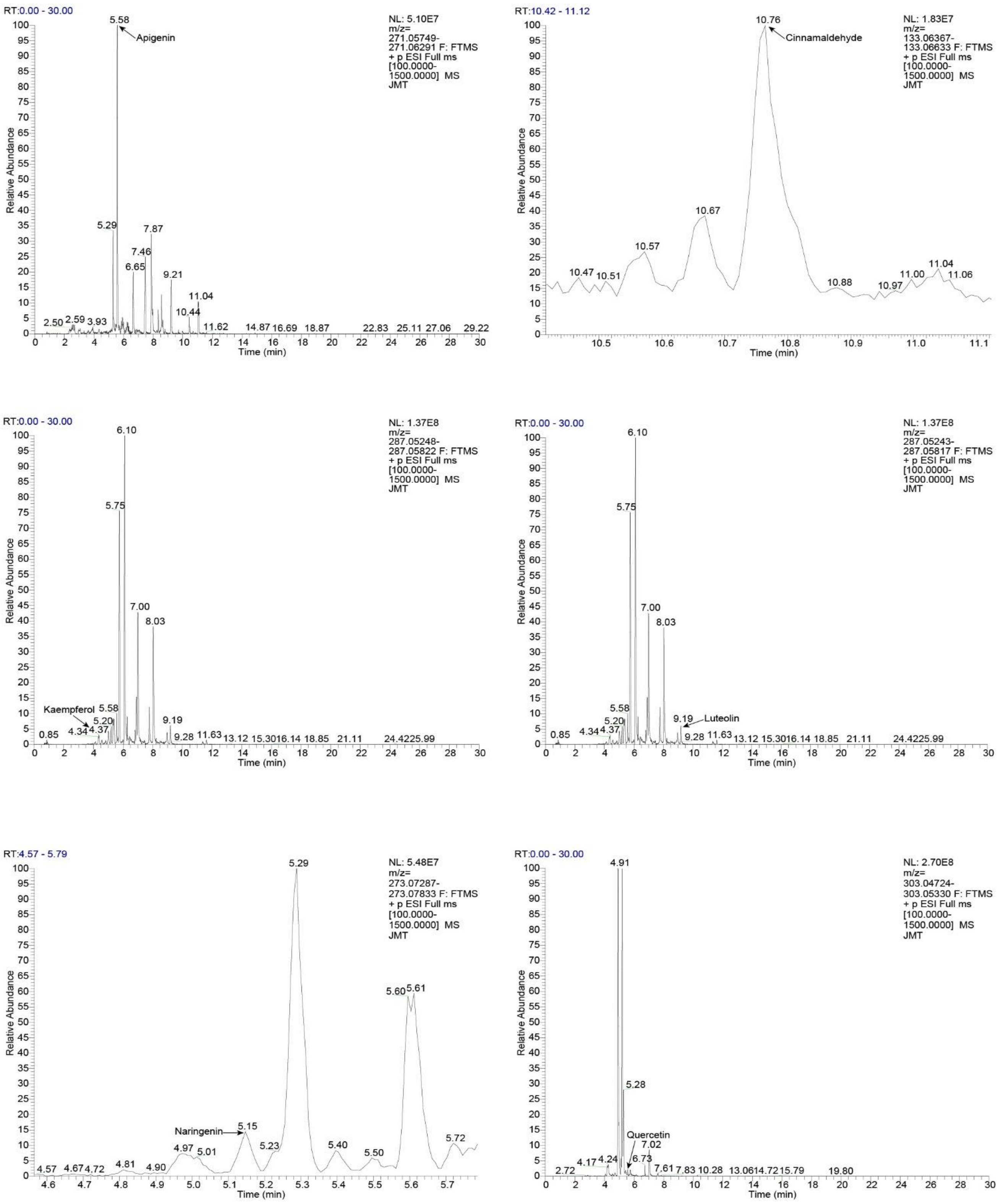

Supplementary Figure 2. The extracted ion chromatograms of six known components in JMT.
